# Tetrandrine enhances anti-PD-1 immunotherapeutic efficacy for hepatocellular carcinoma by activating STING/TBK1/IRF3 pathway

**DOI:** 10.3389/fimmu.2026.1850149

**Published:** 2026-06-09

**Authors:** Biao Zheng, Guojun Yao, Yuli Pang, Jielong Luo, Zhiyong Yang, Fengmin Xiu, Chaohui Zhen, Rui Liang

**Affiliations:** 1 Department of Surgery, The First Dongguan Affiliated Hospital, Guangdong Medical University, Dongguan, Guangdong, China; 2 Department of Geriatrics, The First Affiliated Hospital of Shenzhen University, Shenzhen Second People’s Hospital, Shenzhen, Guangdong, China; 3 Health Management Center, The First Dongguan Affiliated Hospital, Guangdong Medical University, Dongguan, Guangdong, China; 4 Department of Gastrointestinal Surgery, The First Dongguan Affiliated Hospital, Guangdong Medical University, Dongguan, Guangdong, China

**Keywords:** CD8+ T cells, hepatocellular carcinoma, PD-1, STING/TBK1/IRF3 signaling pathway, tetrandrine

## Abstract

**Introduction:**

Hepatocellular carcinoma (HCC) remains a significant global public health risk, and its mortality rate remains high. Tetrandrine (TET) inhibits tumor growth, but its combination with anti-PD-1 immunotherapy has not been fully elucidated.

**Methods:**

STING was knocked down in HCC cells, and CD8^+^ T cells were extracted and activated from peripheral blood. CD8^+^ T cells were co-cultured with HCC cells, and TET was added. This study evaluated TET’s effects on HCC malignant behavior and CD8^+^ T cell activation in the co-culture system. γ-H2AX and dsDNA were detected through immunofluorescence and Western blot. The interaction between TET and the proteins of STING/TBK1/IRF3 pathway was predicted by molecular docking, and the activation of this pathway was analyzed by Western blot. Subcutaneous and orthotopic HCC models were established in mice. The therapeutic effects of TET+anti-PD-1 were evaluated by tumor volume measurement, histopathological analysis and immunohistochemistry, and serum biochemical indicators were detected for safety evaluation.

**Results:**

TET significantly inhibited HCC growth and induced apoptosis. It also promoted CD8^+^ T cell activation, proliferation and cytotoxicity, and enhanced their ability to secrete IFN-γ and TNF-α. TET also induced DNA damage and dsDNA accumulation, and activated STING/TBK1/IRF3 signal. STING knockdown experiments confirmed that this pathway was central for TET anti-cancer effects. In addition, TET and anti-PD-1 produced a significant synergistic anti-tumor effect, effectively inhibited HCC growth, increased CD8^+^ T cell infiltration, and significantly improved liver and kidney function indicators. No obvious toxic reaction was observed.

**Discussion:**

By activating STING/TBK1/IRF3 signaling, TET enhanced CD8^+^ T cell-mediated anti-tumor immunity, thereby markedly enhancing anti-PD-1 therapy efficacy in HCC.

## Graphical Abstract

**Figure:**
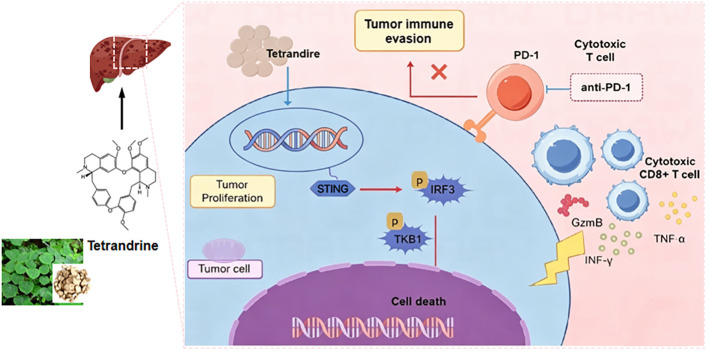
TET inhibits cancer cell malignant biological behavior and enhances CD8^+^ T cell activation via activating STING/TBK1/IRF3 axis, which significantly improves anti-PD-1 therapeutic effects on HCC.

## Highlights:

- Tetrandrine (TET) treatment enhances CD8^+^ T cell activation and enhances the killing effect on cancer cells.
- TET treatment increases the accumulation of cytoplasmic dsDNA.
- TET activates STING/TBK1/IRF3 signaling.
- TET increases CD8^+^ T cell activity and markedly improves anti-PD-1 therapeutic effect on HCC.

## 1 Introduction

As a high-risk malignant tumor, hepatocellular carcinoma (HCC) is insidious and progresses rapidly. Most patients have lost the opportunity of radical surgery at the time of diagnosis, which poses a major threat to human health worldwide (1, 2). Due to the rapid progression of the disease, the prognosis of advanced HCC patient is often poor. The application of traditional treatment methods (liver transplantation) in advanced patients is limited, and the efficacy of chemotherapy and radiotherapy is not satisfactory. Cancer immunotherapy has achieved remarkable success in solid tumor treatment. In particular, immune checkpoint inhibitors (ICI), which revitalize anti-tumor immune responses by restoring tumor-infiltrating T cells (TILs) function (3). Programmed cell death protein 1 (PD-1)/programmed cell death-ligand 1 (PD-L1) inhibitors are most recommended ICI drugs. PD-L1 immune checkpoint inhibitors kill tumor cells by removing tumor cell inhibition on immune system and activating immune cells (4). Nevertheless, few cancer patients respond to ICI therapy (5, 6). Therefore, effective promotion of immune cell infiltration may improve the efficacy of tumor immunotherapy.

Tetrandrine (TET) is a bisbenzylisoquinoline alkaloid extracted from the root of *Stephania tetrandra* S. Moor. It has anti-tumor, anti-inflammatory, anti-allergic, immunosuppressive, calcium channel-blocking, antioxidant, antibacterial and other pharmacological effects (7, 8). It can inhibit the occurrence and development of lung cancer, HCC, prostate cancer and other tumors by inducing apoptosis and autophagy of tumor cells, inhibiting their proliferation, migration, invasion, and reversing multiple drug resistance (9). At present, TET inhibits liver cancer cell metastasis and growth (10, 11). However, whether TET can inhibit HCC by promoting the effect of immunotherapy has not been studied in depth.

The stimulator of interferon gene (STING) is the core hub of innate immunity, which can mediate abnormal DNA immune perception (12) and is key for anti-tumor immune response. In tumors, malignant transformation or treatment will produce cytoplasmic double-stranded DNA (dsDNA), and dsDNA accumulation can activate STING, which is then phosphorylated by downstream TANK binding kinase 1 (TBK1) and interferon regulatory factor 3 (IRF3). A variety of chemokines (such as C-X-C motif chemokine ligand 10 (CXCL10) and C-C motif chemokine ligand 5 (CCL5)) were induced (12). These proteins are important for T cell initiation, thereby enhancing the anti-cancer immune response (13). Activating STING/TBK1/IRF3 signaling can increase T cell infiltration and potentiate PD-1 inhibitors’ anti-tumor effects (14). Studies have shown that radiation-induced DNA damage can drive CD8^+^ T cell chemotaxis via promoting STING signaling pathway, reprogram tumor immune microenvironment, trigger anti-tumor immune response, increase tumor radiation lethality, and promote radiation-mediated tumor regression (15). Since TET increases dsDNA production (16, 17), and TET can increase cytoplasmic dsDNA and activate STING/TBK1/IRF3 signaling, thereby promoting tumor infiltration of macrophages and CD8^+^ T cells and inhibiting tumor growth in non-small cell lung cancer (NSCLC) (18). This study should be noted that while it demonstrated the efficacy of TET in activating the STING pathway in NSCLC, the tumor immune microenvironment of HCC differs substantially from that of NSCLC. HCC is characterized by a more immunosuppressive landscape, lower T cell infiltration, and poorer response rates to immune checkpoint inhibitors. Therefore, it is necessary to validate whether TET exerts similar immunomodulatory effects in HCC and to investigate the underlying mechanisms in this distinct tumor context. Thus, we hypothesized that TET activates this signaling by dsDNA generation to increase anti-PD-1 immunotherapeutic efficacy.

Based on the above background, this study aims to systematically explore whether TET can reshape the immune microenvironment of HCC by activating STING/TBK1/IRF3 signaling, thereby enhancing anti-PD-1 therapeutic effect. This study intends to observe TET’s effects on HCC cell malignant progression, CD8^+^ T cell activation, the accumulation of dsDNA and STING/TBK1/IRF3 axis. To investigate whether TET and anti-PD-1 combination showed a significant synergistic anti-tumor effect on HCC in two C57BL/6 mouse HCC models: subcutaneous transplantation tumor and orthotopic liver tumor. This study not only reveals a new strategy of immune sensitization based on natural drugs, but also provides important experimental basis and possible therapeutic targets for improving immunotherapeutic efficacy for HCC, providing a reference for pharmacological research and clinical application of TET against HCC.

## 2 Methods

### 2.1 Cell grouping and processing

Human fetal hepatocytes THLE-2 and hepatoma cell lines (BNL-CL.2 cells, Hepa1–6 cells and PLC/PRF/5 cells) were purchased from Procell system (CL-0833, CL-0037, CL-0105, CL-0415, Wuhan, China). The cryopreserved cells were thawed in a constant temperature water bath at 37°C, centrifuged, resuspended and quickly inoculated in DMEM medium containing 10% fetal bovine serum (FBS) and 1% penicillin-streptomycin solution (PM150210, Procell system), and cultured at 37°C in a 5% CO_2_ cell incubator (CellXpert C170, Thermo Fisher, USA). The cells were routinely cultured to a density of 80%-90% for subculture for subsequent experiments. All cell lines were identified by STR, and mycoplasma was detected regularly to ensure that the results were negative.

THLE-2 and hepatoma cell lines were exposed to 0, 1, 2, 4, 8 and 12 μM TET (HY-13764, MedChemExpress, Shanghai, China) for 24 h, respectively (18). In addition, some cells were treated with 10 μM cGAS inhibitor RU.521 (HY-114180, MedChemExpress) for 30 min and then treated with TET. 10 μL of CCK-8 working (C0037, Beyotime, Shanghai, China) was added, and incubated in the incubator for 4 h. The optical density (OD) value of cells at 450 nm was measured by a microplate reader (xMark, Bio-Rad, United States) to evaluate cell viability.

Hepa1–6 cells and PLC/PRF/5 cells were exposed to 0, 2, 4 and 8 μM TET for 24 h, respectively, which were recorded as Control group, TET-L group, TET-M group and TET-H group. Then cell viability was determined.

### 2.2 Cell transfection

Short hairpin RNA (Si-STING) and negative control (Si-NC) were purchased from GenePharma (Shanghai, China). Hepa1–6 cells and PLC/PRF/5 cells in logarithmic growth phase were inoculated in 6-well plates, and the above vectors were transfected into cells according to Lipofectamine™ 3000 (L3000001, Invitrogen, USA) instructions. After 24 h, STING knockdown efficiencies were detected using Western blot. Cells were exposed to 8 μM TET.

### 2.3 Colony formation experiment

The cultured HCC cells were digested and resuspended into single cell suspension. The cells were inoculated and cultured for 14 d until cell colonies (>50 μm in diameter) were visible under a microscope. After the culture, the culture medium was discarded, and the cell colonies were fixed with 4% paraformaldehyde (P0099, Beyotime) for 20 min, and then stained with 0.1% crystal violet solution (C0121, Beyotime) for 5 min. Finally, the excess dye was gently washed away with running water, and the culture dish was dried at room temperature. Observe the cell colony (usually defined as a colony with a population of more than 50 cells), count and take photos.

### 2.4 Cell scratch test

HCC cells were cultured and treated accordingly. The 100 μL pipette tip was used to create a straight scratch across the cell monolayer in a straight line. Then, the cell migration of each group was observed at 0, 24 and 48 h after culture, and the relative migration rate of each group was measured and recorded using Image J software.

### 2.5 Transwell invasion assay

Matrigel was thawed overnight at 4°C, placed on an ice table, and mixed with pre-cooled DMEM medium at a ratio of 1: 8. 60 μL Matrigel was added vertically and evenly to the upper chamber to avoid bubble formation. After incubation for 1 h, the matrix gel was discarded and the remaining liquid in the upper chamber was discarded. DMEM medium was added to each hole in the upper and lower chambers, and the membrane was allowed to hydrate for 2 h. HCC cells (2.5×10^5^ cells/mL) were taken, and 150 μL was added to the upper chamber. A total of 600 μL of complete medium containing 20% FBS was added to the lower chamber. After 48 h of culture, the upper and lower chambers were discarded. After washing with PBS, 4% paraformaldehyde was added and fixed for 15 min. The cells were stained with 0.1% crystal violet for 30 min, and rinsed with PBS buffer for 3 times. The non-invasive cells and Matrigel in the upper chamber were gently wiped off with cotton swabs. Five regions were randomly selected for photographing under a microscope and counting.

### 2.6 Cell co-culture

CD8^+^ T cells were isolated from the spleen of C57BL/6 mice using a CD8^+^ T cell isolation kit (19853, STEMCELL, Vancouver, Canada). The spleen was ground to prepare a single cell suspension, and CD8^+^ T cells were isolated by magnetic bead negative selection. The isolated CD8^+^ T cells were cultured in RPMI 1640 medium containing 10% FBS and 10 IU/mL interleukin (IL)-2. The cells were seeded in 6-well plates coated with anti-CD3 (2 μg/mL) and anti-CD28 (1 μg/mL) antibodies (19) and cultured to initiate and maintain CD8^+^ T cell activation and proliferation.

Hepa1–6 cells and PLC/PRF/5 cells were co-cultured with activated CD8^+^ T cells at a ratio of 5: 1, and TET was added for 24 h (20).

### 2.7 CFSE

CD8^+^ T cells (1×10^6^ cells/mL) were resuspended in PBS. Carboxyfluorescein succinimidyl ester (CFSE) working solution was added to a final concentration of 5 μM and incubated in dark for 20 min. Subsequently, 5-fold volume of pre-cooled medium was immediately added to terminate labeling. Then, these CD8^+^ T cells were co-cultured. After culture, CD8^+^ T cell was collected and incubated with CD8 (ab237709, Abcam, USA) flow antibody for 0.5 h. Cells were resuspended and CD8^+^ T cell proliferation level was measured using flow cytometry.

### 2.8 Lactate dehydrogenase assay

CD8^+^ T cell cytotoxicity was tested using LDH kits (C20301, Thermo Fisher). The co-cultured cell culture medium was collected, centrifuged for 10 min, 100 μL supernatant was taken, and 60 μL LDH detection solution was added for incubation in dark for 30 min. The OD_490 nm_ values were read using a microplate reader. Cytotoxicity (%) = (reaction pore OD_490 nm_-natural release pore OD_490 nm_)/(maximum release pore OD_490 nm_-natural release pore OD_490 nm_)×100% (21).

### 2.9 Flow cytometry experiment

HCC cells (1×10^5^ cells) of each group and HCC cells in the co-culture system were taken in a flow tube. Each tube was added with 5 μL PI and Annexin V-FITC (G1511–50 T, Servicebio, Wuhan, China), incubated for 15 min, washed once, resuspended with 500 μL phosphate-buffered saline (PBS). Apoptosis was detected on a flow cytometer (FACSCelesta, BD Biosciences, USA).

### 2.10 Detection of dsDNA by Picogreen method

The content of dsDNA in HCC cells was detected according to the manufacturer’s instructions of the PicoGreen dsDNA fluorescence quantitative determination kit (P7581, Thermo Fisher). After the treatment of HCC cells, the cell lysate was added, the cell membrane was lysed on ice, and the supernatant was collected after centrifugation. Standard samples were prepared at concentrations of 1000, 100, 25, 10, 2.5, and 0.25 ng/mL, and the cell supernatant samples were diluted. 100 μL of diluted PicoGreen working solution was added. The mixture was mixed well and incubated in the dark for 5 min. The signal intensity was detected by a fluorescence microplate reader. The excitation and emission wavelengths were 480 and 520 nm, respectively. The standard curve was drawn and the dsDNA levels of each group were calculated.

### 2.11 Molecular docking

The PDB database was used to obtain the 3D structure files of STING (PDB ID: 6NT5), TBK1 (PDB ID: 7EA2) and IRF3 (PDB ID: 5JER) receptor proteins. The 3D structure file of TET was downloaded from PubChem database. The receptors and ligands were processed by Py MOL 2.5.0 and Auto Dock Tools 1.5.7 software, and the pdbqt format was output. The molecular docking of the receptors and ligands was performed by Auto Dock Vina. The molecular docking data were imported into Py MOL 2.5.0 software for visual display.

### 2.12 Mice grouping and intervention

All animal experiments were approved by the First Dongguan Affiliated Hospital, Guangdong Medical University Ethics Committee (No.:A202201143). Female SPF grade C57BL/6 mice, 6–8 weeks of age, weighing 18–22 g, purchased from Sberfos Biotechnology Co., Ltd. (Beijing, China). All mice were housed in an SPF animal room with 22°C and 50% relative humidity to simulate normal circadian rhythms. Mice had free access to food and water.

In this study, mouse subcutaneous and orthotopic liver tumor models were constructed. Subcutaneous tumor model: 1×10^6^ Hepa1–6 cells were subcutaneously injected into the right lower limb of mice. *In situ* liver tumor model: Hepa1–6 and matrigel were mixed at a volume ratio of 1: 1 to prepare a suspension containing 1×10^6^ Hepa1–6 cells. Then, the midline laparotomy was performed, and the suspension of Hepa1–6 cells was injected into the left hepatic lobe under the liver capsule. After the operation, the abdominal wall was sutured and penicillin was given to prevent infection. After 7 days, when the tumor volume reached 100 mm^3^, the mice were randomly divided into 4 groups: Model group, anti-PD-1 group, TET-H group and TET-H+anti-PD-1 group. Mice in the TET-H group were intraperitoneally injected with 20 mg/kg TET every two days. Mice in the anti-PD-1 group were intraperitoneally injected with 100 μg anti-PD-1 (BE0146, BioXcell, USA) every two days. Mice in the TET-H+anti-PD-1 group were given 20 mg/kg TET and 100 μg anti-PD-1 at the same time for 18 days. During this period, the body weight and tumor diameter were measured every three days, and the tumor volume was calculated following the Pre-proof 11 formula ‘tumor volume = π/6 × length × width^2^’.

After the treatment, mice were weighed and recorded by electronic balance. Subsequently, mice were anesthetized with isoflurane (induction concentration of 3%, maintenance concentration of 1.5%, oxygen flow rate of 1 L/min), and then blood was collected from the retro-orbital sinus, allowed to stand at 4°C for 1.5 h, and then centrifuged. The serum was collected into a centrifuge tube and stored at -20°C. The mice were sacrificed by cervical dislocation under anesthesia to minimize animal suffering, and the heart, liver, spleen, lung, kidney, thymus and tumor tissues in each group were collected and weighed on an electronic balance. The ratio of liver and thymus weight to body weight, namely liver index and thymus index, was calculated. Part of organs and tumor tissues were immersed in 4% paraformaldehyde for fixation. Another part of the tumor was stored at -80°C.

### 2.13 Pathological staining

HE staining: The fixed mouse heart, liver, spleen, lung, kidney, subcutaneous tumor and orthotopic liver tumor tissues were dehydrated by gradient ethanol, transparent by xylene, and embedded in paraffin. The sections were serially sectioned with a slicer (RM2255, Leica, Germany), with a thickness of about 5 μm. Subsequently, HE staining was performed. The steps included hematoxylin staining for 5 min, rinsing with running water to return to blue, eosin staining for 2 min, and neutral gum sealing. The sections were observed under an optical microscope (BX53, Olympus, Japan) and the images were collected for analysis.

TUNEL staining: Paraffin sections of subcutaneous tumors and orthotopic liver tumor tissues were deparaffinized to water, protease K repair antigen without DNase, and membrane rupture solution. Following TUNEL kits (G1504, Servicebio), the TUNEL reaction solution was prepared and added to the slice, and the TUNEL reaction solution was incubated for 1 h. DAPI reaction was added for 10 min. The staining was observed under a fluorescence microscope and the image was taken. Six fields of vision were selected, the average optical density value of apoptotic cells was recorded by Image J software.

### 2.14 Immunohistochemistry

After paraffin sections of subcutaneous tumors and orthotopic liver tumor tissues were baked, sections were deparaffinized with xylene, and gradient ethanol was hydrated. Endogenous peroxidase was inhibited by incubation with 3% H_2_O_2_ for 20 min in the dark. The slices were placed in sodium citrate-citric acid buffer. The slices were heated to above 95°C by microwave oven and kept at high temperature for 20 min, then removed and cooled to room temperature naturally. Ki67 (ab279653, 1: 1000, Abcam) and STING (ab288157, 1: 2000, Abcam) primary antibodies were added, and the refrigerator was kept at 4°C overnight. The first antibody was discarded, PBS was washed, the second antibody (ab6728, 1: 2000, Abcam) was added, incubated for 2 h, DAPI staining, hematoxylin nuclear re-staining. Gradient ethanol dehydration, xylene transparent after sealing. Six random views were selected to take photos and perform data statistics.

### 2.15 Immunofluorescence

HCC cells in each group were fixed with 4% paraformaldehyde. The cells were stained with PicoGreen dsDNA fluorescent reagent and examined by a fluorescence microscope. HCC cells and mouse tumor tissues were fixed with 4% paraformaldehyde and blocked with 10% goat serum for 30 min. Phosphorylation of histone H2AX (γ-H2AX, 2577, 1: 1000, Cell Signaling Technology, USA), PD-L1 (ab213480, 1: 200, Abcam), CD4 (ab288724, 1: 50, Abcam), CD8 (ab316778, 1: 100, Abcam) antibodies were added and incubated overnight at 4°C. After that, fluorescent antibody IgG and DAPI staining were added and incubated for 2 h and 10 min, respectively. The slides were dried and sealed with anti-fluorescence quencher. The slides were observed under a fluorescence microscope. The protein fluorescence intensity was evaluated using Image J software.

### 2.16 Western blot

The mouse subcutaneous tumor and *in situ* tumor tissue were quickly thawed, and HCC cells in each group were collected, and RIPA lysate containing protease inhibitors (G2002, Servicebio) was added to homogenize. After lysis and centrifugation, the supernatant contained the total protein. Then the protein was quantified by BCA kit (G2026, Servicebio), and the corresponding sample volume was calculated. After protein denaturation, SDS-PAGE gel was prepared. After gel electrophoresis separation, the protein was transferred to PVDF membrane, and the membrane was transferred to PVDF membranes for 60 min at a constant current of 250 mA at 4°C. The PVDF membrane was blocked with blocking solution (5% skim milk powder) for 90 min, incubated with primary antibodies (**Table 1**), and slowly shaken overnight in a refrigerator at 4°C. The membranes were washed three times with TBST and incubated with the corresponding species secondary antibody for 2 h. The ECL luminescent chromogenic solution (G2161, Servicebio) was added dropwise. The gel imaging system (Chemi Doc XRS, Bio-Rad, USA) was used to take pictures. The gray value of protein bands was analyzed by ImageJ software, and different protein expressions were calculated.

**Table 1:** The primary antibodies used for Western blot.

Protein name	Dilution ratio	Catalog number	Producer
B-cell lymphoma 2 (Bcl-2)	1: 1000	ab241548	Abcam
Bcl-2-associated X protein (Bax)	1: 500	ab243140	Abcam
Caspase-3	1: 2000	ab184787	Abcam
Cleaved Caspase-3 (Cl-Caspase-3)	1: 500	ab32042	Abcam
Perforin	1: 1000	ab256453	Abcam
Granzyme B	1: 1000	ab255598	Abcam
cytotoxic T-lymphocyte antigen-4 (CTLA-4)	1: 1000	ab237712	Abcam
PD-L1	1: 2000	DF6526	Affinity
γ-H2AX	1: 1000	2577	Cell Signaling Technology
STING	1: 2000	DF12090	Affinity
p-STING	1: 2000	AF7416	Affinity
TBK1	1: 5000	ab40676	Abcam
p-TBK1	1: 2000	AF8190	Affinity
IRF3	1: 1000	ab68481	Abcam
p-IRF3	1: 2000	AF2436	Affinity
CCL5	1: 2000	AF5151	Affinity
CXCL10	1: 2000	DF6417	Affinity
GAPDH	1: 10000	ab181602	Abcam

### 2.17 ELISA and biochemical examination

The co-cultured cell culture medium was transferred to a sterile centrifuge tube, and the supernatant was collected after centrifugation. At the same time, the serum of each group was taken. The levels of interferon-γ (IFN-γ, SEKH-0046, Solarbio) and tumor necrosis factor-α (TNF-α, SEKH-0047, Solarbio) were measured by ELISA. The mixture of supernatant or serum and antibody was incubated in an ELISA plate for 1 h. 100 μL of the substrate was added and incubated 10 min. Then 100 μL of the termination reaction solution was added to detect the OD values and evaluate IFN-γ and TNF-α content.

Alanine aminotransferase (ALT), aspartate aminotransferase (AST), blood urea nitrogen (BUN), γ-glutamyl transferase (γ-GT) and alkaline phosphatase (ALP) kits were purchased from Nanjing Jiancheng Bioengineering Institute (C009-2-1, C010-2-1, C013-2-1, C017-2-1, A059-2-2, Nanjing, China). The serum of subcutaneous tumor and orthotopic tumor mice was taken and the experiment was carried out following the manufacturer’s instructions. The OD values of each well were detected by microplate reader. The standard curve was prepared and the OD values were taken to calculate concentration.

### 2.18 Statistical analysis

Statistical analysis was conducted using SPSS version 27.0. Normality and homogeneity of variance were assessed for all data sets. One-way ANOVA was utilized, followed by Tukey’s *post hoc* test for group comparisons. For evaluating the synergistic effect of TET and anti-PD-1 in animal studies, two-way ANOVA was performed to assess the interaction between TET and anti-PD-1 treatment. Data for each group were presented as mean ± standard deviation, with *P* < 0.05 deemed statistically significant.

## 3 Results

### 3.1 TET significantly inhibited HCC cell malignant progression

**Figure 1:**
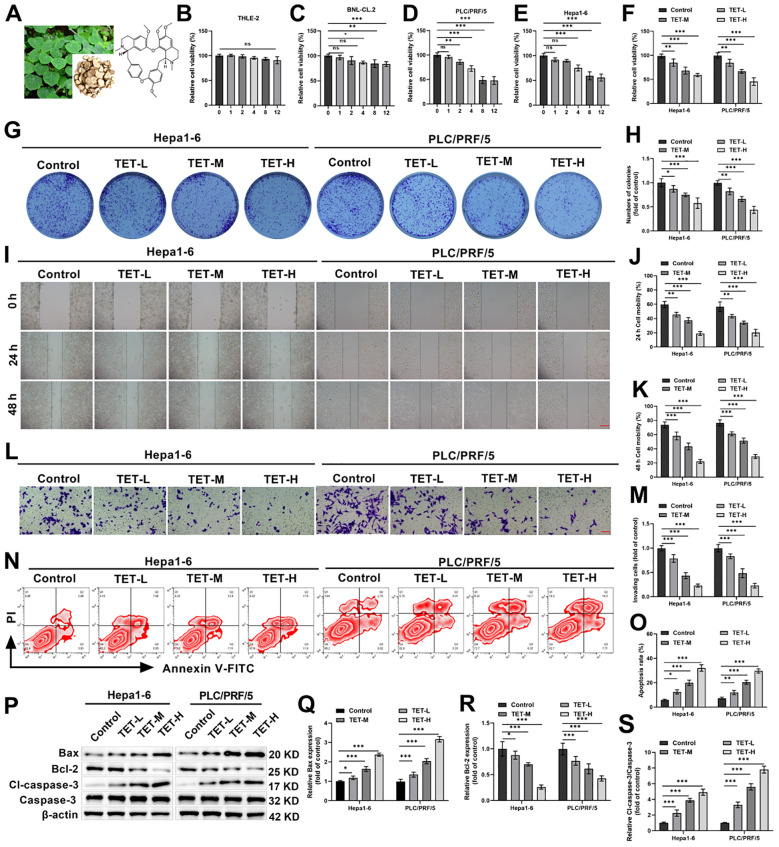
TET significantly inhibited HCC cell malignant progression. **(A)** *Stephania tetrandra* S. Moor pictures and TET structural formula. **(B–E)** THLE-2, BNL-CL.2, PLC/PRF/5 and Hepa1–6 cells were treated with 0-12 μM TET for 24 h, and cell viability was detected by CCK-8. TET significantly reduced the viability of HCC cell lines. **(F)** PLC/PRF/5 and Hepa1–6 cells were treated with 2, 4 and 8 μM TET for 24 h, and CCK-8 measured cell viability. **(G, H)** The colony formation ability was observed, which was significantly decreased after TET intervention. **(I–K)** Cell scratch assay detected cell migration. TET significantly reduced HCC cell migration rate (×20, 100 μm). **(L, M)** Transwell assay measured cell invasion. TET significantly reduced invasive cell number (×20, 100 μm). **(N, O)** Flow cytometry examined cell apoptosis. It increased significantly when TET intervention. P-S: Western blot detected apoptotic proteins. TET markedly enhanced Bax and Cl-Caspase-3 expressions, and declined Bcl-2 level. n=6, ns*P* > 0.05 *P < 0.05, **P < 0.01, ***P < 0.001.

TET is a bisbenzylisoquinoline alkaloid (**Figure 1A**), which can inhibit the development of various tumors. In this study, the impacts of TET on HCC cells were systematically evaluated. TET (1-12 μM) had no statistically significant impact on THLE-2 cell viability. TET markedly suppressed the viability of HCC cell lines, and the inhibitory effect on PLC/PRF/5 and Hepa1–6 cells was particularly obvious (**Figures 1B–E**). Therefore, these two cells were selected as experimental cell lines, and 2, 4 and 8 μM were used as the intervention doses of TET. After that, HCC cells were exposed to 2, 4 and 8 μM TET. TET inhibited HCC cell viability (**Figure 1F**), reduced the number of cell colonies (**Figures 1G, H**), and significantly reduced the colony formation ability. TET also markedly suppressed the cell migration rate at 24 and 48 h (**Figures 1I–K**) and reduced the number of invasive cells (**Figures 1L, M**). These results indicated that TET treatment effectively suppressed HCC cell growth, movement and invasion. Moreover, TET significantly increased the apoptosis rate of HCC cells (**Figures 1N, O**). Western blot results further confirmed that TET could up-regulate Bax and Cl-Caspase-3 expressions, and down-regulate Bcl-2 level (**Figures 1P–S**), indicating that TET induced apoptosis of HCC cells by regulating apoptosis proteins. In summary, TET could effectively inhibit HCC cell malignant progression *in vitro*.

### 3.2 TET treatment enhanced CD8^+^ T cell activation and enhanced the killing effect on cancer cells

**Figure 2:**
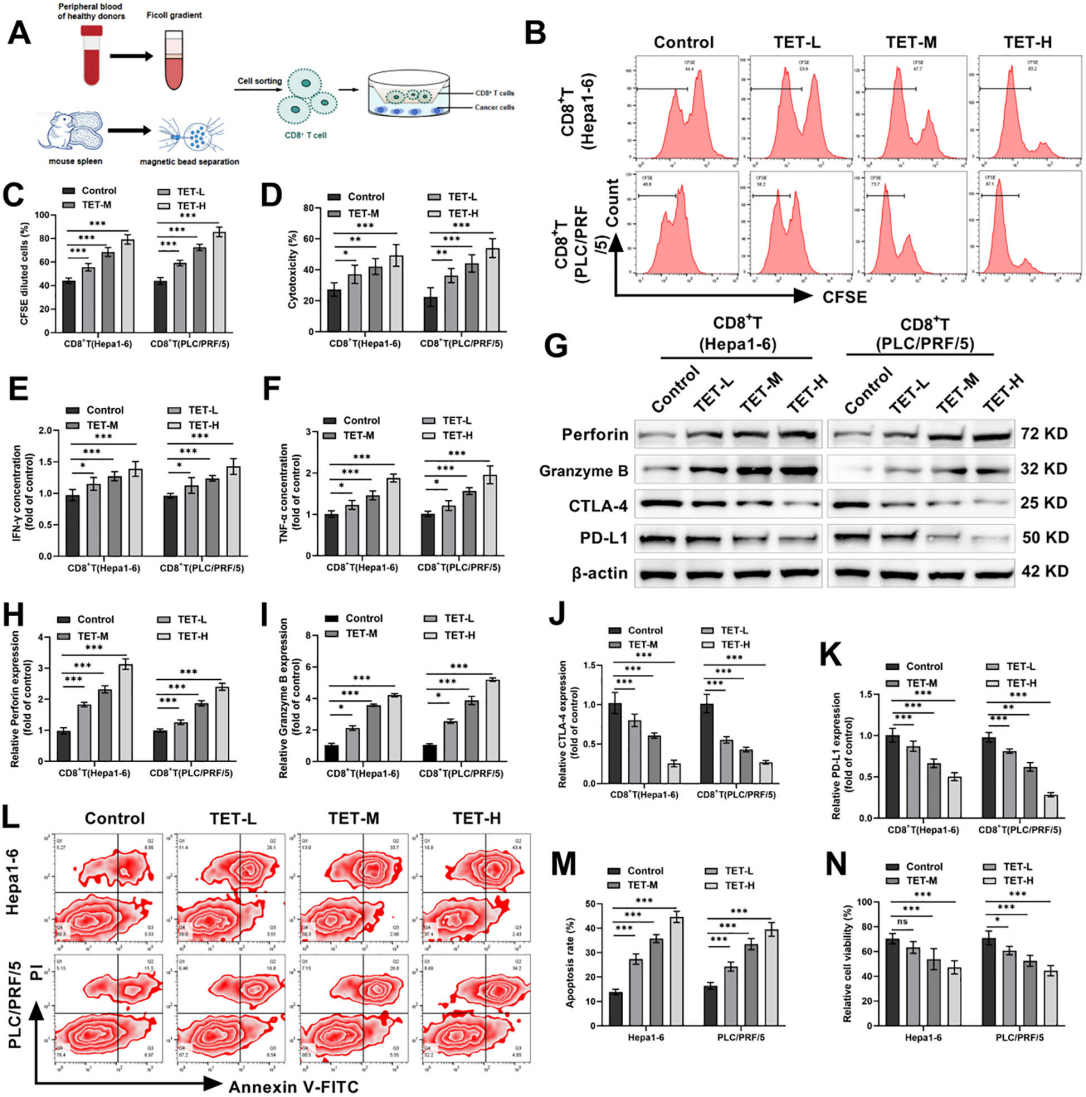
TET treatment enhanced CD8^+^ T cell activation and enhanced the killing effect on cancer cells. **(A)** *In vitro* process diagram of co-culture of CD8^+^ T cells and hepatoma cells. **(B, C)** CFSE assay detected CD8^+^ T cell proliferation, which was markedly increased when TET treatment. **(D)** LDH kits examined CD8^+^ T cell cytotoxicity, which was significantly increased after TET treatment. **(E, F)** IFN-γ and TNF-α were examined by ELISA, which were significantly increased after TET treatment. **(G–K)** Western blot detected tumor immune-related proteins. TET markedly enhanced the expression of Perforin and Granzyme B, and significantly reduced CTLA-4 and PD-L1 protein levels. **(L, M)** Flow cytometry examined HCC cell apoptosis in co-culture system. It increased significantly after TET intervention. **(N)** CCK-8 method examined HCC cell viability in the co-culture system. It was significantly reduced after TET intervention. n=6, *P < 0.05, **P < 0.01, ***P < 0.001.

This study further explored the role of TET in tumor immunotherapy. First, CD8^+^ T cells were extracted and activated, then co-cultured with HCC cells, and TET was used for intervention. The experimental process was shown in **Figure 2A**. In tumor immunotherapy, CD8^+^ T cell proliferation is one of immune activation direct markers. TET markedly promoted the proliferation of CD8^+^ T cells (**Figures 2B, C**). LDH release assay confirmed that TET enhanced CD8^+^ T cell cytotoxic effects on HCC cells (**Figure 2D**). IFN-γ and TNF-α secreted by CD8^+^ T cells are the core indicators reflecting the killing ability of tumor cells. After TET treatment, their contents were significantly increased (**Figures 2E, F**). TET significantly increased CD8^+^ T cell activation marker Perforin and Granzyme B expressions, and significantly reduced CTLA-4 and PD-L1 expressions (**Figures 2G–K**). TET significantly increased HCC cell apoptosis rate in the co-culture system (**Figures 2L, M**), and significantly reduced the cell viability (**Figure 2N**), indicating that TET treatment enhanced CD8^+^ T cell killing effects on HCC cells, resulting in a decrease in cancer cell viability. Based on the above results, TET effectively activated CD8^+^ T cells, enhanced their proliferation, activation and killing ability to HCC cells.

### 3.3 TET increased cytoplasmic dsDNA accumulation

**Figure 3:**
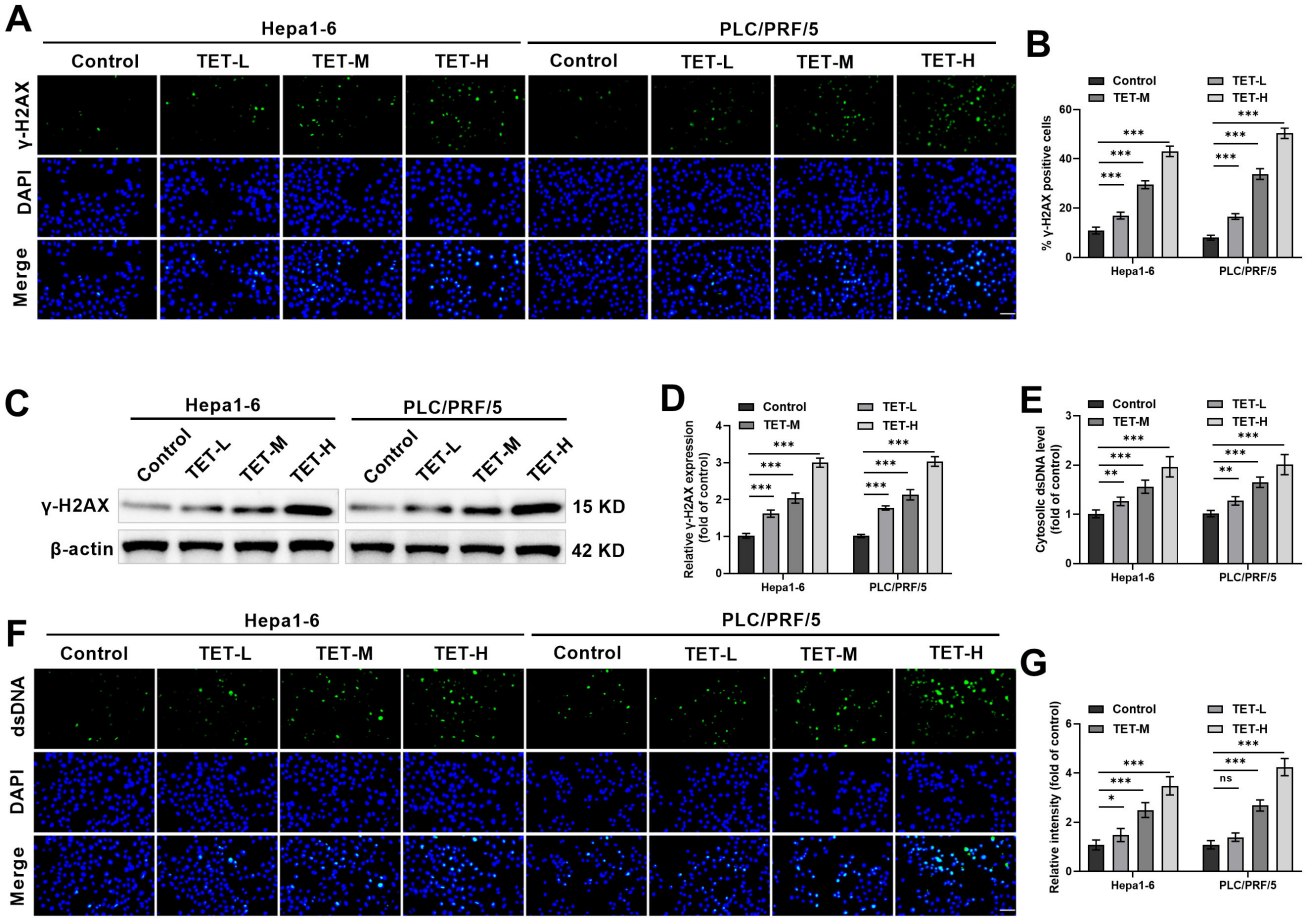
TET increased cytoplasmic dsDNA accumulation. **(A, B)** Immunofluorescence examined DNA damage marker γ-H2AX formation. TET significantly increased γ-H2AX positive cells (×40, 50 μm). **(C, D)** Western blot detected γ-H2AX protein, which was significantly increased after TET treatment. **(E)** Picogreen dsDNA fluorescence quantification was detected after 24 h of TET treatment, which was significantly increased after TET treatment. **(F, G)** dsDNA content was detected by immunofluorescence. TET significantly increased dsDNA content (×40, 50 μm). n=6, ns*P* > 0.05 *P < 0.05, **P < 0.01, ***P < 0.001.

We detected γ-H2AX expression. γ-H2AX positive cell percentage was markedly enhanced after TET therapy (**Figures 3A, B**). And γ-H2AX protein level also increased significantly after TET treatment (**Figures 3C, D**). This indicated that TET caused significant DNA damage. Further analysis of Picogreen dsDNA fluorescence quantification and immunofluorescence showed that the content of dsDNA in the cytoplasm was significantly increased after TET intervention (**Figures 3E–G**). These results collectively indicated that TET induced DNA injury of HCC cells and led to cytoplasmic dsDNA accumulation.

### 3.4 TET promoted STING/TBK1/IRF3 axis

**Figure 4:**
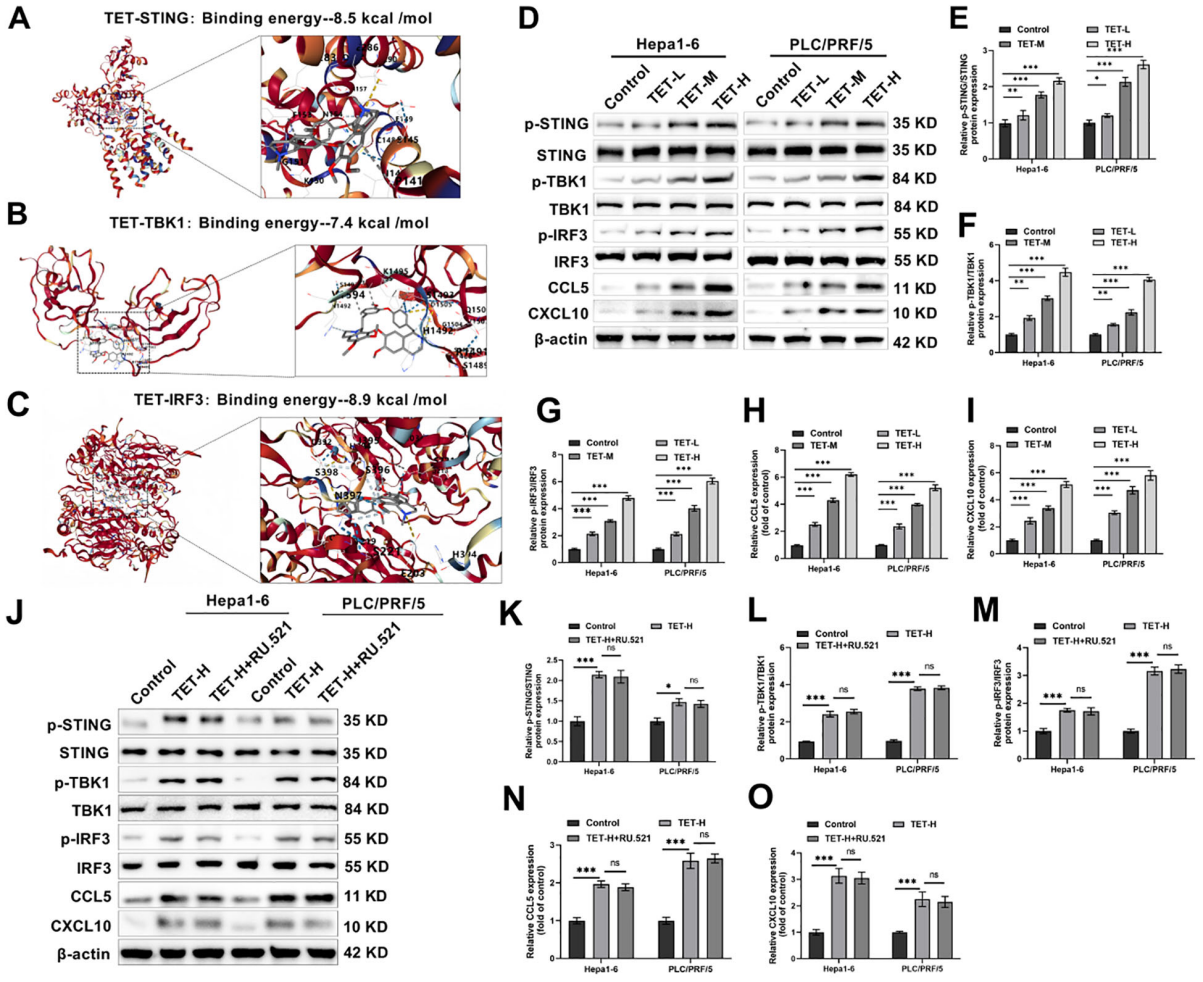
TET promoted STING/TBK1/IRF3 axis. **(A–C)** Molecular docking detected the affinity of TET with STING, TBK1 and IRF3, respectively. **(D–I)** Western blot examined STING/TBK1/IRF3 pathway protein level. TET markedly enhanced p-STING, p-TBK1, p-IRF3, CCL5 and CXCL10 proteins. **(J–O)** Hepa1–6 and PLC/PRF/5 cells were treated with TET and cGAS inhibitor RU.521, and western blot examined STING/TBK1/IRF3 pathway protein level. RU.521 did not affect the STING/TBK1/IRF3 pathway proteins. n=6, ns*P* > 0.05, *P < 0.05, **P < 0.01, ***P < 0.001.

STING/TBK1/IRF3 signaling is vital for cancer disease development. Exploring the potential mechanisms of TET treatment on HCC, we first performed molecular docking analysis. The results showed that TET exhibited predicted binding energies with STING, TBK1 and IRF3, suggesting potential interactions that may affect protein function or activation status. The binding energies of TET with STING, TBK1 and IRF3 were -8.5, -7.4 and -8.9 kcal/mol, respectively (**Figures 4A–C**). These predicted binding energies suggest potential interactions that warrant further investigation. Further Western blot experiments confirmed this finding. In TET-treated HCC cells, phosphorylated STING, TBK1 and IRF3 protein levels were significantly raised, and the expression of its downstream chemokines CCL5 and CXCL10 also increased (**Figures 4D–I**). In order to determine whether the activation of TET through the STING pathway depends on the upstream cGAS sensor, we used the cGAS inhibitor RU.521 for combined intervention on the basis of TET treatment. The results showed that TET+RU.521 treatment did not significantly eliminate TET-induced phosphorylation of STING, TBK1 or IRF3, and did not affect the up-regulation of CCL5 and CXCL10 (**Figures 4J–O**). These results suggest that TET may activate the STING/TBK1/IRF3 signaling axis through a mechanism that appears to be at least partially independent of cGAS, although the involvement of cGAS cannot be completely excluded based solely on RU.521 inhibition. The above results indicated that TET could activate the STING/TBK1/IRF3 signaling and its downstream immune response, although the precise molecular mechanism remains to be elucidated.

### 3.5 TET activated STING/TBK1/IRF3 signaling to inhibit HCC cell malignant biological behavior and promote CD8^+^ T cell activation

**Figure 5:**
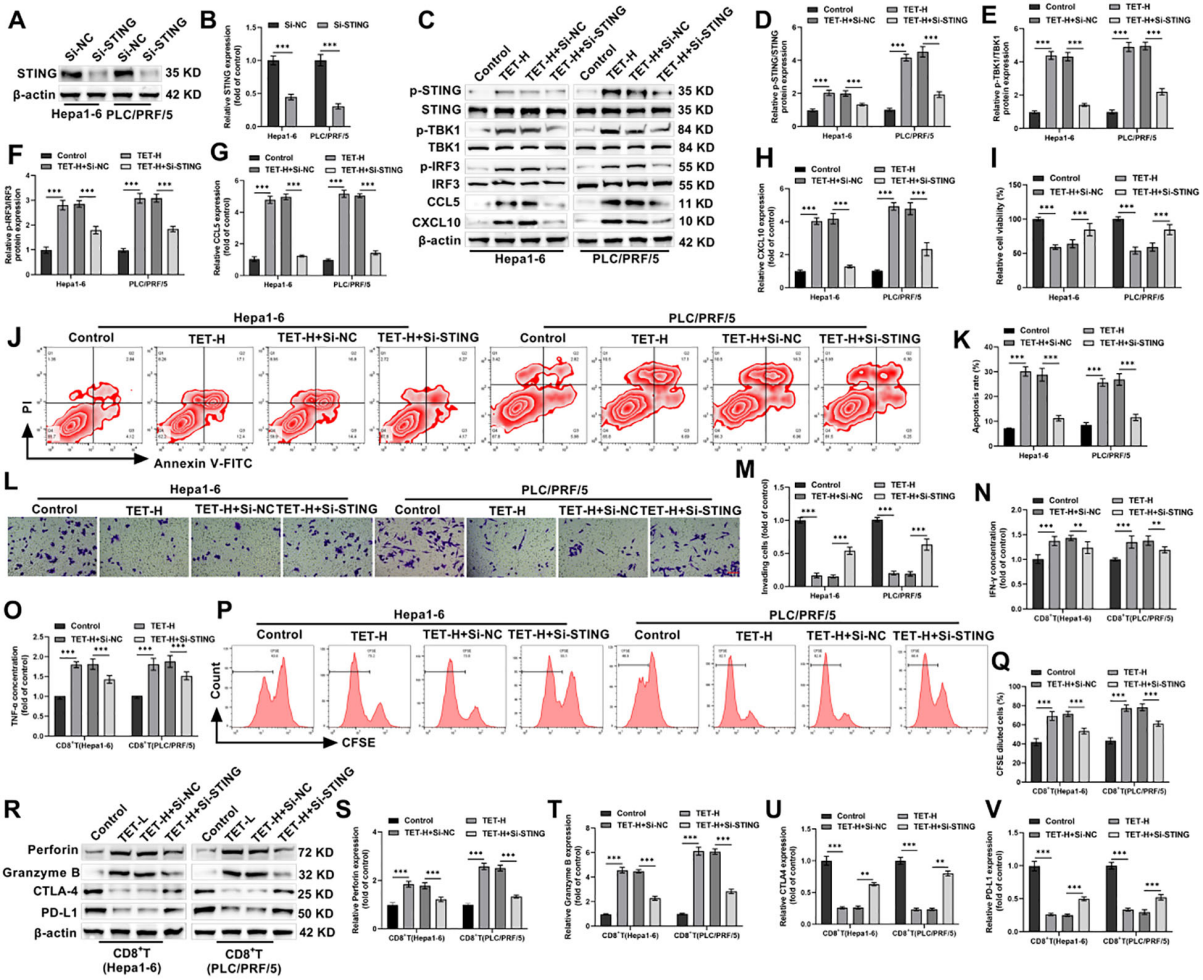
TET activated STING/TBK1/IRF3 signaling to inhibit HCC cell malignant biological behavior and promote CD8^+^ T cell activation. **(A, B)** Si-STING and Si-NC were transfected into HCC cells. The knockdown efficiency of STING was examined using Western blot. **(C–H)** Western blot detected STING/TBK1/IRF3 pathway protein. Knockdown of STING significantly reduced p-STING, p-TBK1, p-IRF3, CCL5 and CXCL10 proteins. **(I)** CCK-8 detected Hepa1–6 and PLC/PRF/5 cell viability, which was significantly increased after STING knockdown. **(J, K)** Flow cytometry examined PLC/PRF/5 and Hepa1–6 cell apoptosis. It was significantly reduced after STING knockdown. **(L, M)** Transwell assay detected cell invasion. Knockdown of STING markedly increased invasive cell number (×20, 100 μm). **(N, O)** IFN-γ and TNF-α were examined by ELISA, which were significantly reduced after STING knockdown. **(P, Q)** CFSE assay detected CD8^+^ T cell proliferation, which was markedly declined when STING knockdown. **(R–V)** Western blot detected tumor immune-related proteins. Knockdown of STING markedly reduced CD8^+^ T cell activation markers Perforin and Granzyme B expressions, and significantly increased CTLA-4 and PD-L1 expressions. n=6, **P < 0.01, ***P < 0.001.

This study verified that the core role of TET depends on STING/TBK1/IRF3 signaling activation via knocking down STING in HCC cells. Si-STING and Si-NC were transfected into cells. Si-STING significantly reduced STING protein levels (**Figures 5A, B**), suggesting that STING was successfully knocked down. After STING knockdown, TET-induced phosphorylation of STING, TBK1 and IRF3 and the up-regulation of downstream chemokines CCL5 and CXCL10 were significantly reversed (**Figures 5C–H**), suggesting that STING/TBK1/IRF3 signaling was significantly inhibited. Compared to TET intervention, STING knockdown significantly increased HCC cell viability (**Figure 5I**), reduced the apoptosis rate (**Figures 5J, K**), and increased the number of invasive cells (**Figures 5L, M**). STING knockdown effectively offset the inhibitory effects of TET on HCC cell malignant progression. In immune co-culture system, STING knockdown also weakened CD8^+^ T cell activation by TET, which was manifested in significant reduction of CD8^+^ T cell proliferation (**Figures 5P, Q**), cytotoxicity (IFN-γ and TNF-α secretion) (**Figures 5N, O**) and Perforin and Granzyme B expressions, while increasing PD-L1 and CTLA-4 expressions (**Figures 5R–V**). These results demonstrated that TET inhibited HCC cell malignant biological behavior and promoted CD8^+^ T cell immune responses by activating STING/TBK1/IRF3 signaling.

### 3.6 Inhibition of TET combined with anti-PD-1 monoclonal antibody on tumor growth

**Figure 6:**
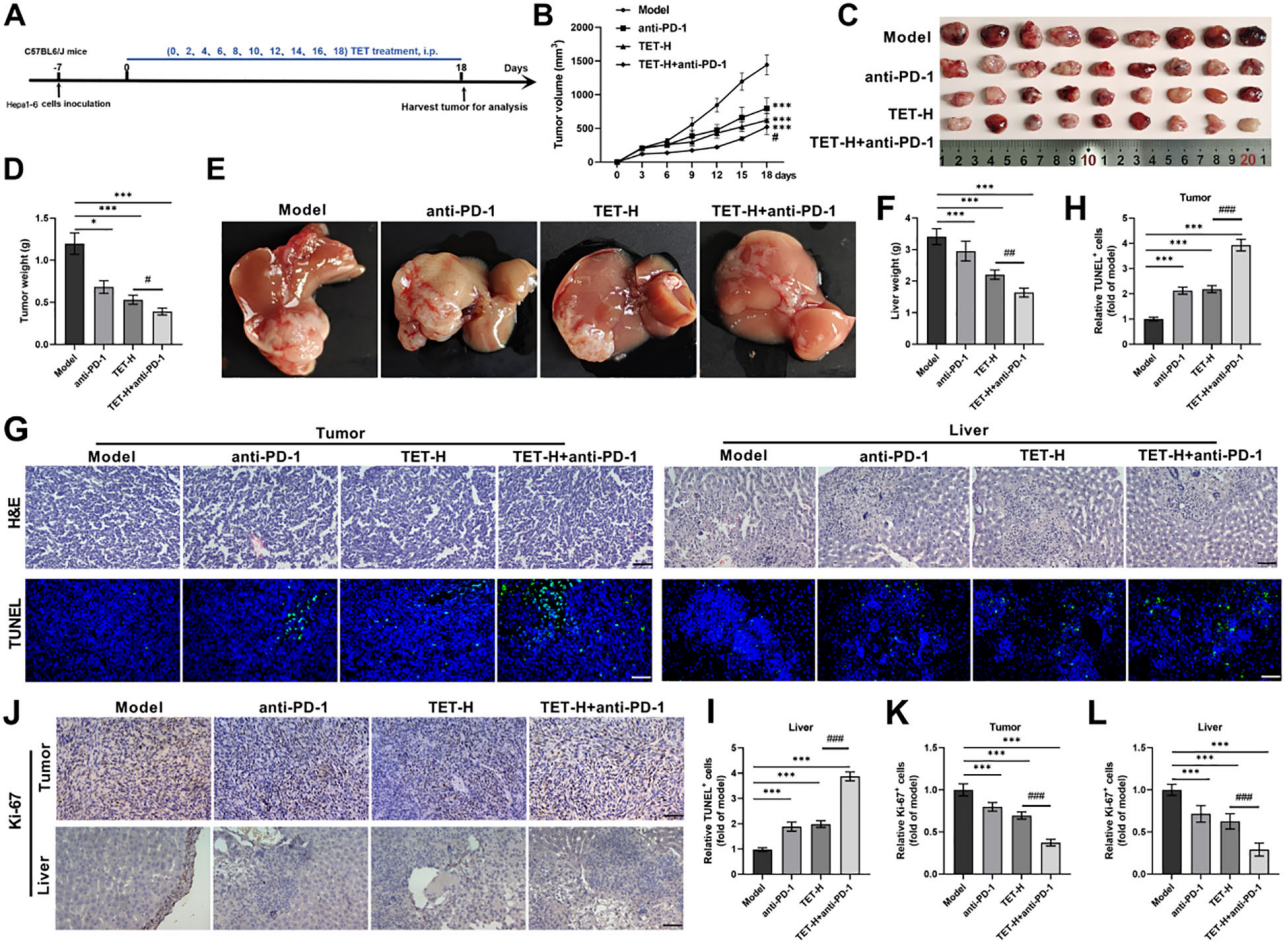
Inhibitory effect of TET combined with anti-PD-1 on cancer proliferation. **(A)** Tumor construction and treatment diagram. **(B–D)** Morphological image, subcutaneous transplanted tumor volume and weight in mice. TET treatment significantly reduced the volume and weight of subcutaneous xenografts. **(E, F)** Morphological images and tumor weight of orthotopic liver tumors in mice. TET treatment significantly reduced the weight of orthotopic liver tumors. **(G)** HE staining images of subcutaneous xenografts and orthotopic liver tumors in mice. TET intervention resulted in necrotic areas in tumor tissues (×40, 50 μm). **(H, I)** TUNEL staining representative images and histograms of subcutaneous xenografts and orthotopic liver tumors in mice. TET treatment significantly increased the rate of TUNEL-positive cells (×40, 50 μm). **(J–L)** Ki67 immunohistochemical representative images and histograms of subcutaneous transplanted tumors and orthotopic liver tumors in mice. TET treatment markedly lessened the proportion of Ki67 positive cells (×40, 50 μm). n=6, *P < 0.05, ***P < 0.001. #*P* < 0.05, ##*P* < 0.01, ###*P* < 0.001

Two C57BL/6 mouse models of subcutaneous transplanted tumor and orthotopic liver tumor were constructed by injecting the suspension of Hepa1–6 cells. Tumor mice were intraperitoneally injected with TET and anti-PD-1 every 2 days. Tumor model construction and treatment schematic diagram was shown in **Figure 6A**. TET and anti-PD-1 treatment markedly reduced subcutaneous xenografts volume and weight (**Figures 6B–D**) and reduced orthotopic liver tumor weight (**Figures 6E, F**), and TET+anti-PD-1 was the most effective at inhibiting cancer growth. HE staining showed that after TET and anti-PD-1 intervention, large and dense necrotic areas appeared in subcutaneous xenografts and orthotopic liver tumor tissues (**Figure 6G**). TUNEL staining found that TET and anti-PD-1 significantly raised the TUNEL-positive cell rate in these two tumor tissues (**Figures 6H, I**) and significantly reduced Ki67-positive proliferating cells (**Figures 6J–L**). Moreover, TET+anti-PD-1 had the most significant therapeutic impact. The combined treatment induced the most extensive apoptosis, and the proliferation activity was most effectively inhibited. These *in vivo* experiments found that TET effectively inhibited tumor growth, had a synergistic effect with anti-PD-1 therapy, which greatly enhanced its therapeutic effect on HCC.

### 3.7 TET enhanced the activity of CD8^+^ T cells via activating STING/TBK1/IRF3 pathway and significantly improved anti-PD-1 therapy efficacy on HCC

**Figure 7:**
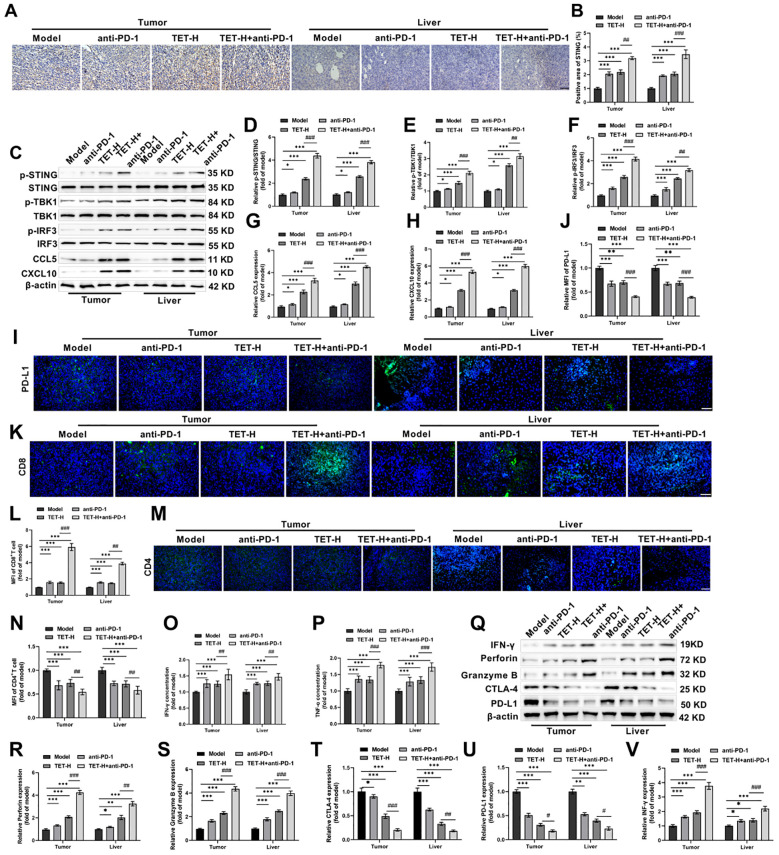
TET enhanced CD8^+^ T cell activity via activating STING/TBK1/IRF3 pathway and significantly improved anti-PD-1 therapy efficacy on HCC. **(A, B)** STING immunohistochemical representative images and histograms of subcutaneous xenografts and orthotopic liver tumors in mice. TET treatment significantly increased STING positive area (×40, 50 μm). **(C–H)** Western blot examined STING/TBK1/IRF3 pathway in subcutaneous xenografts and orthotopic liver tumors. TET significantly increased p-STING, p-TBK1, p-IRF3, CCL5 and CXCL10 proteins. **(I, J)** IFN-γ and TNF-α contents were detected using ELISA, which were significantly raised after TET and anti-PD-1 treatment. **(K–P)** PD-L1, CD4 and CD8 immunofluorescence representative images and histograms of subcutaneous transplanted tumors and orthotopic liver tumors. TET intervention significantly reduced the fluorescence intensity of PD-L1 and CD4, and significantly increased the fluorescence intensity of CD8 (×40, 50 μm). **(Q–V)** Western blot detected tumor immune-related proteins within tumor tissues. TET treatment significantly increased Perforin, Granzyme B and IFN-γ expressions, and significantly reduced CTLA-4 and PD-L1 proteins. n=6, *P < 0.05, **P < 0.01, ***P < 0.001. ##*P* < 0.01, ###*P* < 0.001

Having confirmed that TET enhanced CD8^+^ T cell activity *in vitro*, we validated these findings *in vivo*. STING positive area in subcutaneous xenografts and orthotopic liver tumors increased significantly after TET and anti-PD-1 intervention, and the STING positive area was the largest after TET and anti-PD-1 combined intervention (**Figures 7A, B**). The expression levels of key proteins in STING/TBK1/IRF3 pathway (p-STING, p-TBK1, p-IRF3) and their downstream chemokines CCL5 and CXCL10 were markedly up-regulated when TET treatment, but anti-PD-1 only significantly increased p-TBK1 and p-IRF3 protein levels (**Figures 7C–H**). IFN-γ and TNF-α contents of subcutaneous tumor and orthotopic tumor model mice increased significantly after TET and anti-PD-1 treatment, and the most significant increase after combined treatment (**Figures 7I, J**). These changes in cytokines might be due to CD8^+^ T cell infiltration. Therefore, immunofluorescence analysis further found that TET and anti-PD-1 treatment significantly enhanced CD8^+^ T cell infiltration proportion, and significantly reduced the fluorescence intensity of PD-L1 and CD4^+^ T cell infiltration (**Figures 7K–P**), indicating that the immune response was enhanced. Perforin and Granzyme B expressions, markers of CD8^+^ T cell activation, were enhanced, while CTLA-4 and PD-L1 expressions were inhibited, and these protein levels were significantly higher in the combined treatment group (**Figures 7Q–U**). It was worth noting that the expression level of IFN-γ protein was significantly increased after TET and anti-PD-1 treatment (**Figure 7V**). Compared with single therapy, TET+anti-PD-1 had a more obvious anti-cancer effect. TET promoted CD8^+^ T cell activation and infiltration through activating STING/TBK1/IRF3 signaling, thereby significantly increasing anti-PD-1 therapeutic effects on HCC.

### 3.8 *In vivo* safety evaluation of TET combined with anti-PD-1 immunotherapy

**Figure 8:**
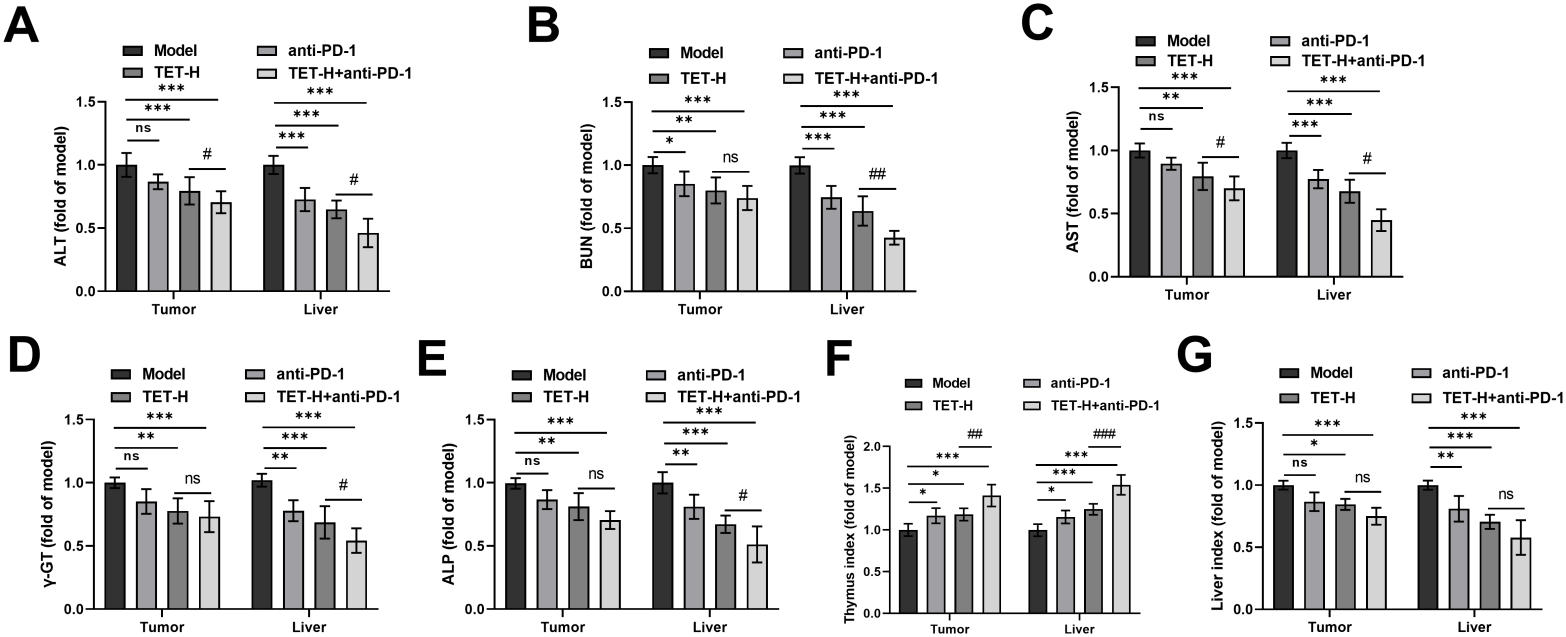
*In vivo* safety evaluation of TET combined with anti-PD-1 immunotherapy. **(A–E)** The levels of liver and kidney function indexes in serum of subcutaneous tumor mice and *in situ* tumor mice were detected by kits. ALT, AST, BUN, γ-GT and ALP contents were markedly declined after combined TET and anti-PD-1 therapy. **(F, G)** Liver and thymus index of subcutaneous tumor mice and orthotopic tumor mice. After combined TET and anti-PD-1 therapy, the thymus index increased significantly and the liver index decreased significantly. n=6, *P < 0.05, **P < 0.01, ***P < 0.001. ns*P* > 0.05, #*P* < 0.05, ##*P* < 0.01, ###*P* < 0.001

We systematically analyzed the main organs and serum biochemical indicators. The serum biochemical analysis showed that ALT, AST, BUN, γ-GT and ALP contents with subcutaneous xenografts and orthotopic liver tumors were significantly reduced after combined TET and anti-PD-1 therapy (**Figures 8A–E**), suggesting that this treatment regimen effectively alleviated liver and kidney function damage associated with tumor models. In addition, after combined TET and anti-PD-1 therapy, the thymus index increased significantly and the liver index decreased significantly, reflecting the improvement of immune function and the reduction of tumor load, respectively (**Figures 8F, G**). These results suggested that TET combined with anti-PD-1 treatment had protective effects on tumor-induced multiple organ function injury.

## 4 Discussion

Although the immunotherapy of HCC has made great progress, the response rate of anti-PD-1 monotherapy is still not ideal. Exploring effective combined sensitization strategies is the top priority of current research. Many literatures have confirmed that TET has a strong anti-cancer effect (22, 23). This study systematically revealed that TET activated STING/TBK1/IRF3 signaling, reshaped immunological microenvironment, and promoted CD8^+^ T cell activation and infiltration, thus significantly enhancing anti-PD-1 therapeutic effect on HCC.

Many studies have shown that cell proliferation is a vital target for cancer treatment (24). Metastasis is a cellular biological process that involves a cascade of sequential migration and invasion, allowing cancer cells to spread to other tissues and organs. As cancer advances to metastasis, it greatly diminishes the survival rate for patients, with approximately 90% ultimately passing away (25). We first confirmed the direct anti-tumor activity of TET on HCC cells, including inhibition of growth, migration and invasion and induction of apoptosis, which is consistent with the previous understanding of the anti-cancer effects of TET (26, 27).

In addition, CD8^+^ T cells are important cells for cancer immunity and are the first line of defense against tumor cells. Their enhanced infiltration indicates that effective anti-tumor immune response may be produced. Activated CD8^+^ T cells can kill tumor cells by secreting toxic factors such as TNF-α, IFN-γ, Perforin and Granzyme B (28). However, in chronic diseases such as cancer, CD8^+^ T cell function is in a state of disorder. Tumors can obtain immune escape by inhibiting CD8^+^ T cell infiltration and activating or inducing dysfunction by immunoediting the tumor microenvironment to promote malignant tumor progression (29, 30). As an important immune checkpoint molecule, PD-L1 can interact with PD-1 to suppress CD8^+^ T cell proliferation and facilitate cancer cells to escape from the immune system (31, 32). CTLA-4 is expressed on T cells (33). After T cell activation, CTLA-4 is highly expressed and competitively binds to CD28, blocking T cell activation and inhibiting immune function (34). In this study, CD8^+^ T cells were co-cultured with PLC/PRF/5 and Hepa1–6 cells in the same system, and TET intervention observed CD8^+^ T cell activation and its effects on the killing of HCC cells. TET significantly promoted CD8^+^ T cell activation, proliferation and killing function. TNF-α, IFN-γ, Perforin and Granzyme B contents were markedly enhanced, and CTLA-4 and PD-L1 expressions were significantly lessened. This indicated that TET enhanced CD8^+^ T cell immune function. TET might be one of the potential effective drugs for HCC therapy.

However, this study did not stop there, but further explored the potential mechanism of its regulation of tumor immune microenvironment. Our key finding was that TET treatment effectively induced DNA damage in HCC cells and led to a significant accumulation of cytoplasmic dsDNA. STING can bind to 2’,3’-cyclic GMP-AMP dinucleotide to form a polymer, which is then transferred to the Golgi apparatus to recruit the kinase TBK1 and phosphorylate it, and then promote the phosphorylation of IRF3, stimulate the production of downstream factors, activate the body ‘s innate immune system (35), and then exert anti-tumor effects. Immunity mediated by the STING/TBK1/IRF3 signaling is vital for inhibiting tumor development (36). Shi et al. found that fucoidan MF4 could also inhibit Lewis lung cancer through activating STING/TBK1/IRF3 signaling (37). Zhang et al. showed that Qianlie Xiaozheng formula could activate STING/TBK1/IRF3 axis and promote CD8^+^ T cell recruitment, thereby inhibiting prostate cancer (38). Enhancing the activity of this pathway can inhibit the malignant progression of ovarian cancer (39). TET had predicted binding energies with STING, TBK1 and IRF3 proteins, and subsequent Western blot experiments confirmed that TET indeed activated STING/TBK1/IRF3 signaling and promoted downstream chemokines CCL5 and CXCL10 expression, and the higher the TET concentration, the more obvious the corresponding protein up-regulation trend. Unexpectedly, RU.521 treatment failed to significantly block TET-induced activation of the STING/TBK1/IRF3 signaling axis, indicating that TET may activate the STING/TBK1/IRF3 axis through a mechanism that is at least partially independent of cGAS. Therefore, we propose that TET may target the core proteins in the STING/TBK1/IRF3 pathway to exert its activating effect. We observed that DNA damage and cytoplasmic dsDNA accumulation may be a concomitant phenomenon of TET, or as a synergistic factor rather than the main activation mechanism. Our results demonstrated that TET treatment significantly upregulated the expression of CXCL10 and CCL5, two key chemokines downstream of the STING/TBK1/IRF3 pathway. CXCL10 is a potent chemoattractant for activated CD8^+^ T cells and natural killer (NK) cells via its receptor CXCR3 (40). Similarly, CCL5 recruits T cells, dendritic cells, and monocytes through CCR5 (41–43). The upregulation of these chemokines in the tumor microenvironment is critical for breaking immune tolerance and converting “cold” tumors into “hot” tumors. Our co-culture experiments further confirmed that TET-treated HCC cells promoted CD8^+^ T cell migration and infiltration, consistent with the increased chemokine expression. Therefore, we propose that TET not only activates the STING pathway but also creates a chemokine gradient that facilitates CD8^+^ T cell recruitment to the tumor site, thereby potentiating anti-PD-1 immunotherapy. This chemotactic effect represents an important mechanism by which TET enhances T cell-mediated antitumor immunity. Subsequently, we speculated that TET might inhibit the malignant biological behavior of HCC cells and enhanced the immune function of CD8^+^ T cells via activating STING/TBK1/IRF3 axis. This experiment conducted a recovery experiment by knocking down STING. Knockdown of STING significantly promoted PLC/PRF/5 and Hepa1–6 cell proliferation, inhibited CD8^+^ T cell activation and proliferation and their killing function on HCC cells. Inhibition of TET on HCC cell malignant phenotype and CD8^+^ T cell activation were dependent on STING/TBK1/IRF3 signaling activation. Moreover, studies have found that down-regulation of STING/TBK1/IRF3 signaling reduces IFN-γ and Perforin contents, thereby reducing the activity of natural killer cells (44), STING/TBK1/IRF3 signaling could play killing effects on cells through regulating IFN-γ and Perforin levels. STING/TBK1/IRF3 signaling activation through TET enhanced the immune function of CD8^+^ T cells, it may be related to changes in IFN-γ and Perforin.

It should be noted that in **Figure 5C**, the total protein level of STING in the TET-H + Si-STING group was similar to that in the Control group and the TET-H group. This phenomenon may have the following explanation: First, **Figures 5A, B** shows the results of verifying the STING knockdown efficiency without TET treatment, while **Figures 5C–H** shows the protein expression level after 18 hours of TET treatment. TET may up-regulate the expression of STING protein through transcriptional or post-translational mechanisms, thus partially offsetting the knockdown effect of Si-STING. This compound-induced compensatory up-regulation of target proteins is not uncommon in drug treatment combined with gene knockdown experiments. Secondly, and more importantly, the core finding of **Figures 5C–H** is not the knockdown efficiency of STING total protein, but the TET-induced downstream signals (p-STING, p-TBK1, p-IRF3, CCL5, CXCL10) were significantly reversed after STING knockdown. This result fully demonstrates that the activation of downstream signals by TET depends on the presence of STING. As for whether TET directly binds to STING protein or regulates its phosphorylation level and pathway activity through indirect mechanisms (such as regulating STING upstream signaling molecules, affecting STING post-translational modification or changing its subcellular localization), it is still unclear, and more in-depth mechanism research is needed to clarify it.

A recent study by Tan and colleagues demonstrated that TET activates the STING/TBK1/IRF3 pathway to enhance anti-PD-1 immunotherapy in NSCLC (18). Our study extends these findings to HCC and provides several novel insights. First, while Tan et al. focused on the downstream type I interferon response, we further investigated the upstream mechanism of STING activation and found that TET activates this pathway through a mechanism that appears to be at least partially independent of cGAS, as the cGAS inhibitor RU.521 failed to block TET-induced signaling. Second, we uncovered a cell-intrinsic tumor suppressor function of STING in HCC cells, demonstrating that STING knockdown alone promotes HCC cell proliferation, survival and invasion, a phenomenon not reported in NSCLC. Third, we established both subcutaneous and orthotopic liver tumor models to better recapitulate the HCC immune microenvironment, and systematically evaluated the safety profile of TET+anti-PD-1 synergistic therapy. Collectively, our study not only validates the role of TET as a STING pathway agonist in a different cancer type but also reveals novel mechanistic insights (cGAS independence and cell-intrinsic STING function) that may have implications for TET-based immunotherapy across multiple cancer types.

It is worth noting that this study found that STING knockdown itself could significantly promote the proliferation, inhibit apoptosis and enhance invasion of HCC, suggesting that STING had an intrinsic tumor suppressor function in HCC. Previous studies have shown that the STING pathway can directly regulate the malignant behavior of tumor cells through a variety of mechanisms. STING activation can induce apoptosis through the mitochondrial pathway, involving the regulation of Bcl-2 family proteins and the activation of Caspase cascade (45, 46). Secondly, STING can induce cell cycle arrest through p53-dependent or p53-independent pathways (47). In addition, STING activation can also reduce cell invasion and metastasis by inhibiting epithelial-mesenchymal transition (EMT)-related transcription factors (such as Snail and Twist) (48). In this study, we observed that the expression of Bax and Cl-Caspase-3 was up-regulated and the expression of Bcl-2 was down-regulated after TET treatment, and STING knockdown could reverse these changes, suggesting that STING-mediated apoptosis induction is one of the important mechanisms of its intrinsic tumor suppressor effect. However, whether STING directly inhibits the malignant behavior of HCC cells by regulating cell cycle or EMT-related proteins still needs further experimental verification.

In subcutaneous xenograft and orthotopic liver tumor models, the combined treatment of TET and anti-PD-1 markedly suppressed cancer proliferation, induced cancer cell apoptosis, and significantly promoted CD8^+^ T cell activity and infiltration, remodeling cancer immune microenvironment. TET also stimulated STING/TBK1/IRF3 signaling. It was worth noting that the safety evaluation showed that TET+anti-PD-1 alleviated the abnormal liver and kidney function indexes of tumor-bearing mice to a certain extent. This feature is crucial for the clinical transformation of TET, because many synthetic STING agonists are effective, but often accompanied by severe systemic inflammatory reactions and other side effects (49, 50). As a natural compound, TET shows a better therapeutic window.

Our results showed that TET treatment significantly reduced the expression of two major immune checkpoint molecules, CTLA-4 on T cells and PD-L1 on tumor cells. CTLA-4 competes with CD28 for binding to CD80/CD86 on antigen-presenting cells, delivering an inhibitory signal that dampens early T cell activation (51). PD-L1 engagement with PD-1 on T cells suppresses T cell proliferation, cytokine production, and cytotoxic function (52). The TET-induced downregulation of CTLA-4 and PD-L1 likely relieves this dual immunosuppressive blockade, allowing CD8^+^ T cells to become fully activated and exert potent antitumor effects. Notably, this occurred in the context of elevated IFN-γ levels, which typically upregulates PD-L1 via the JAK/STAT pathway (53, 54). The decoupling of IFN-γ from PD-L1 upregulation may reflect a unique property of TET as a multi-target compound, potentially interfering with IFN-γ receptor signaling or promoting rapid clearance of PD-L1^+^ tumor cells. Collectively, these findings suggest that TET enhances T cell immunity through a dual mechanism: (1) activating the STING pathway to promote chemokine-dependent T cell recruitment, and (2) downregulating immune checkpoints to prevent T cell exhaustion. This combined effect likely underlies the synergistic antitumor activity of TET in combination with anti-PD-1 therapy. This study also found an interesting phenomenon that after TET/anti-PD-1 synergistic therapy, although IFN-γ levels were significantly increased, PD-L1 expression was significantly decreased. According to the classic adaptive immune resistance paradigm, IFN-γ secreted by activated T cells usually up-regulates the expression of PD-L1 in tumor cells through the JAK/STAT signaling pathway, as a negative feedback mechanism to limit excessive immune response. However, we observed the uncoupling of this classical relationship in this study. It is worth noting that a recent study reported a similar finding that increased IFN-γ levels were accompanied by decreased PD-L1 expression in effective combined immunotherapy (18). We speculate that TET, as a multi-target natural compound, may interfere with the JAK/STAT signaling axis downstream of the IFN-γ receptor while activating the STING pathway, thereby relieving the induction of PD-L1 by IFN-γ. In addition, effective synergistic therapy may reduce PD-L1 levels by rapidly removing PD-L1-positive tumor cells or changing the composition of the tumor microenvironment. The precise molecular mechanism of this phenomenon needs to be further elucidated.

Of course, there are still some limitations. First, the effect of TET on other immune cells (such as dendritic cells, regulatory T cells, macrophages) require further investigation. Secondly, this study mainly depends on the mouse model. The pharmacokinetic characteristics of TET in human body and the clinical efficacy of TET combined with anti-PD-1 still need to be verified by clinical trials. Thirdly, TET has calcium channel-blocking activity, which may have an impact on the cardiovascular system. However, this study only evaluated liver and kidney function indicators, and did not specifically evaluate the cardiovascular system. Although no obvious behavioral abnormalities or death were observed in mice at the dose of this study, subclinical cardiovascular function changes cannot be completely ruled out. Follow-up studies will supplement the safety assessment of the cardiovascular system to comprehensively evaluate the safety characteristics of TET combined with anti-PD-1 therapy. In addition, although molecular docking predicted potential interactions between TET and STING/TBK1/IRF3 proteins, which might influence their phosphorylation and activation. They were only computational predictions and did not necessarily reflect real biophysical interactions. Therefore, these predictions still need to be verified by direct binding experiments such as surface plasmon resonance (SPR) or cellular thermal shift assay (CETSA). Fifth, the conclusion regarding the cGAS-independent mechanism of TET-induced STING activation is preliminary and requires further validation. The experiments using the cGAS inhibitor RU.521 alone are not sufficient to completely rule out cGAS involvement. Future studies using cGAS knockout cells or more specific genetic approaches are needed to confirm this finding. Therefore, in the current manuscript, we present this observation as a hypothesis rather than a definitive conclusion.

## 5 Conclusion

In summary, TET recruits and activates CD8^+^ T cells by activating the STING/TBK1/IRF3 signaling pathway, and ultimately significantly enhances the efficacy of anti-PD-1 immunotherapy for HCC. TET-induced DNA damage and cytoplasmic dsDNA accumulation may be a secondary phenomenon rather than the main mechanism. Our work not only provides novel joint strategies in overcoming the resistance of HCC immunotherapy, but also provides a strong theoretical basis and experimental support for mining immunotherapy sensitizers from natural products of traditional Chinese medicine. TET is expected to become an immunotherapy adjuvant with clinical application prospects.

## Data Availability

The original contributions presented in the study are included in the article/supplementary material. Further inquiries can be directed to the corresponding authors.
